# Rufinamide-Loaded Chitosan Nanoparticles in Xyloglucan-Based Thermoresponsive *In Situ* Gel for Direct Nose to Brain Delivery

**DOI:** 10.3389/fphar.2021.691936

**Published:** 2021-06-21

**Authors:** Avantika Dalvi, Punna Rao Ravi, Chandra Teja Uppuluri

**Affiliations:** Department of Pharmacy, BITS-Pilani, Hyderabad, India

**Keywords:** chitosan nanoparticles, Rufinamide, thermoresponsive gel, nose to brain delivery, pharmacokinetics

## Abstract

In 2004, the US FDA approved Rufinamide, an anti-epileptic drug under the brand name Banzel^®^. In 2015, Banzel^®^ received approval for its use in pediatric patients (ages 1–4 years). Rufinamide shows low oral bioavailability due to a low dissolution rate resulting in less of the drug reaching the brain. This has led to the high dose and dosing frequency of Rufinamide. In this work, using the principle of design of experiments (DoE), we have formulated Rufinamide-loaded chitosan nanoparticles and suspended them in a solution of a thermoresponsive polymer–tamarind seed xyloglucan to form a nasal *in situ* gel for direct nose to brain delivery of Rufinamide. The nanoparticles were characterized for particle size, entrapment efficiency, zeta potential, and physical stability. The *in situ* gel formulations were characterized for rheological properties, stability, and *in vivo* plasma and brain pharmacokinetics. Pharmacokinetic parameters were computed for aqueous suspension of nanoparticles and *in situ* gelling formulation for nanoparticles and compared with the pharmacokinetic parameters of an aqueous suspension of plain Rufinamide. The percentage of direct transport efficiency (% DTE) and direct transport percentage (%DTP) values were calculated for all the formulations. The optimized nanoparticle formulation showed a size of 180 ± 1.5 nm, a zeta potential of 38.3 ± 1.5 mV, entrapment efficiency of 75 ± 2.0%, and drug loading of 11 ± 0.3%. The *in situ* gelling formulation of nanoparticles showed a solution to the gel transition temperature of 32°C. The %DTE values for aqueous suspension of nanoparticles and *in situ* gelling formulation for nanoparticles were 988.5 and 1177.3 and the %DTP values were 86.06 and 91.5 respectively.

## Introduction

Management of epilepsy syndrome has been a challenge for a long time. In a 2018 analysis, childhood epilepsy was found to be the most frequently occurring chronic neurological disorder in children, affecting 0.5–1.0% of children worldwide. Lennox Gastaut Syndrome (LGS) is a form of childhood epilepsy occurring at ages 3–5, which manifests in adulthood (Aaberg et al., 2017). Patients with LGS often have reduced life expectancy which is mostly attributed to poor seizure control and injuries from falls (MedlinePlus 2020).

Rufinamide (Rufi), a triazole derivative, was approved by the US FDA and EMA for the treatment of LGS in children and adults. Rufi is available as tablets (100–800 mg) and oral suspension under the brand name Inovelon^®^ and Banzel^®^. Rufi is well absorbed when given orally, but the rate of absorption is slow due to its low solubility and slow rate of dissolution of the drug in GI fluids. Since Rufi is prescribed in children from ages 1 and above and given its moderately low oral bioavailability, it is imperative to look for an alternative route that will increase the molecules’ distribution to the brain (Perucca et al., 2008; EMA 2011).

For more than a decade, researchers have started to explore the nose to brain (N2B) route for delivering therapeutics to the brain. N2B is a noninvasive and much more practical method of delivering drugs to the CNS than compared to intrathecal, intraparenchymal, intravenous, and intracerebroventricular injections (Illum 2000). Several formulation strategies such as hydrogels, lipid-based formulations, and particulate formulations (nanoparticles and microparticles) have been explored for the N2B delivery of molecules (Giunchedi et al., 2020).

In our previous work, we evaluated the N2B uptake of the intranasally administered aqueous suspension of Rufi and a thermoresponsive *in situ* gel of Rufi (Dalvi et al., 2021). The formulations showed ∼31 and ∼90% direct nose to brain uptake, respectively. As an extension to our previous work, we wanted to evaluate and compare the N2B uptake of two formulations—aqueous suspension containing Rufi nanoparticles and thermoresponsive nasal *in situ* gel loaded with Rufi nanoparticles.

Nanoparticles for Rufi were prepared using chitosan, a natural, biodegradable polymer. Chitosan-based formulations have been shown to be applicable in N2B transport. Chitosan is a cationic polymer that consists of randomly arranged groups of 2-amino-2-deoxy-D-glucose (D-glucosamine) and 2-acetamido-2-deoxy-D-glucose (N-acetyl-D glucosamine). Chitosan is soluble in aqueous media which have a pH of less than 6. Extensive ability to form intra and intermolecular hydrogen bonding and its cationic nature are responsible for its bio-adhesive properties (Saikia and Gogoi 2015; Aderibigbe and Naki 2019). Several reports over the last decade have shown that chitosan can be used to deliver drugs effectively via the nose to brain pathway. Formulations containing chitosan have shown longer residence time in the nose due to the strong bio-adhesion of chitosan and mucin. Chitosan has also exhibited permeation-enhancing properties intranasally (Aderibigbe and Naki 2019; Rassu et al., 2016).

In this research work, we have developed and optimized Rufi-loaded chitosan–tri polyphosphate nanoparticles using the principles of design of experiments (DoE). Nanoparticles were characterized for their particle size, zeta potential, entrapment efficiency, and drug loading efficiency. Further, the optimized nanoparticles were suspended in water and an *in situ* gel. Rheological analysis was carried out on optimized nanoparticles loaded in the *in situ* gelling vehicle to determine its solution to gel (T_sol→gel_) temperature (Dalvi et al., 2021). *In vivo* studies were conducted to evaluate mucociliary clearance time, plasma and brain pharmacokinetic studies, and nasal toxicity of optimized nanoparticles loaded in the *in situ* gelling formulation as compared to the aqueous suspension of optimized nanoparticles.

## Materials and Methods

### Materials

Rufinamide (Rufi) was obtained from Glenmark Pharmaceuticals, India. Piribedil (Internal Standard, IS) was a gift sample from Reddy’s Laboratories, Hyderabad, India. Tamarind seed xyloglucan (TSX) was a gift sample from Encore Natural Polymers Pvt. Ltd., Ahmedabad, India. β-Galactosidase from Aspergillus oryzae was purchased from Sigma Aldrich, Mumbai, India. Medium molecular weight chitosan (190,000–310,000 Da; 75–85% deacetylated), sodium tri polyphosphate (STPP), mannitol, poloxamer 407, polyethylene glycol 400, and N-methyl-2–pyrrolidone were obtained from Sigma Aldrich, Mumbai, India. HPLC grade methanol, acetonitrile (ACN), glacial acetic acid (GAA), ammonium acetate, and thiomersal were procured from SRL Chemicals Pvt. Ltd., Mumbai, India. For all experimental processes and analysis, HPLC grade Milli Q water from our in-house water purification unit (Millipore^®^, MA, United States ) was used. Male Wistar rats were procured from Veeba Biosciences Pvt. Ltd., Hyderabad, India.

### Preparation of Rufi-Loaded Chitosan Nanoparticles

Rufi-loaded chitosan nanoparticles (Rufi-Ch-NPs) were prepared using the ionic gelation method. First, the solutions of Rufi, STPP, and chitosan were prepared separately. Chitosan solution (1.0%w/v) was prepared by medium molecular weight chitosan in 1.5%v/v of GAA in water. For the preparation of Rufi solution, 5 mg of Rufi and poloxamer 407 were dissolved in 300 µL of NMP followed by the addition of PEG 400 (volume as per the design). STPP solution (30 mg/ml) was prepared by dissolving STPP in water. STPP solution (volume as per the design) was added to the Rufi solution to form a mixture which was then added to the chitosan solution under high-speed homogenization (Polytron PT 3100D, Kinemetica, Lucerne, Switzerland) at room temperature, using a syringe in a dropwise manner. The amount of chitosan (25 mg) taken was kept the same in all the experimental runs. The volume of STPP solution added in the preparation of nanoparticles varied based on the level of chitosan:STPP mass ratio required in the experimental run. Following the homogenization process, the dispersion was subjected to ultrasonication (Vibra cell, Sonics, Connecticut, United States). In the optimization runs, the amplitude (20%) and pulse (3 s on followed by 3 s off) of ultrasonication and homogenization time were kept at fixed values while the homogenization speed was varied according to the design. The dispersion containing Rufi-Ch-NPs obtained after processing was subsequently centrifuged and washed three times to remove the free drug. The nanoparticle pellet was re-dispersed in Milli Q water containing 3.0%w/v mannitol as a cryoprotectant (3.0%w/v of the total volume used to re-disperse the nanoparticles) and freeze-dried (Coolsafe 110–4, Scanvac, Lynge, Denmark). The freeze-dried formulation was stored in air-tight glass vials at 2–8°C until further use.

### Experimental Design for Preparation of Rufi-Ch-NPs

Rufi-Ch-NPs were designed based on the principles of DoE. Particle size (PS) (nm), zeta potential (mV), and entrapment efficiency (EE) (%) of the nanoparticles were identified as critical quality attributes or responses. Critical factors/variables affecting the PS and EE of nanoparticles were first identified using a minimum resolution (Minires) screening design. A total of six factors were screened at two levels [minimum (−1) and maximum (+1)]: A-chitosan: STPP mass ratio (1 and 10), B-volume of PEG 400 (200 and 1,200 µL), C-amount of poloxamer 407 (2 and 10 mg), D-homogenization speed (5,000 and 15,000 rpm), E-homogenization time (5 and 15 min), and F-ultrasonication time (2 and 8 min). A few preliminary trials combined with knowledge from previously reported literature helped in the selection of the polymers, solvents, processing conditions, and their respective high and low levels used in the screening design. The Minires design consisted of 17 runs including 3 center points*.* From the results of the Minires design, four factors *viz.* A-chitosan: STPP mass ratio, B-volume of PEG 400, C-amount of poloxamer 407, and D-homogenization speed, were found to be significantly affecting the responses.

Further, a high-resolution (Box Behnken Design) BBD was applied to understand how these critical factors and their interactions impacted the responses, and to optimize the preparation of Rufi-Ch-NPs. The BBD is a type of response surface design, used to obtain a second-order polynomial equation to optimize a formulation by performing very few experiments. The BBD consisted of 27 runs (inclusive of 5 center runs to check reproducibility) for four factors. The second-order polynomial equation generated from the BBD is of the following form:

$$Y=\beta_{0}+\beta_{1}X_{1}+\beta_{2}X_{2}+\beta_{3}X_{3}+\beta_{12}X_{1}X_{2}+\beta_{23}X_{2}X_{3}+\beta_{13}X_{1}X_{3}+\beta_{11}X_{1}^{2}+\beta_{22}X_{2}^{2}+\beta_{33}X_{3}^{2}$$ (1)

Where, Y is the dependent variable, $\beta_{0}$ is the arithmetic mean response of the 27 runs, ${\beta^{\prime }}_{i}s$ and ${\beta^{\prime }}_{ii}s$ (i = 1–3) are coefficients of individual linear and quadratic effects of the factors, respectively, and ${\beta^{\prime }}_{ij}s$ (i,j = 1–3; i < j) are coefficients of the effect of interaction between the $i^{th}$ and $j^{th}$ factors.

### Desirability Function Model Validation

The optimum values for all factors were given by the Design-Expert version 11 software (Stat-Ease Inc., Minneapolis, MN) based on the desirability function criteria. The desirability function was calculated based on the constraints set for the dependent variables: Maximizing the EE % and zeta potential, and minimizing the PS. In order to validate the model, *n = 6* repetitions were performed based on the experimental conditions predicted by the optimization model. PS, zeta potential, and EE % were determined for all the repetitions. Wilcoxon signed-rank test was performed to check if there was any significant difference between observed values and values predicted by the model.

### Preparation of Rufi-Ch-NPs Loaded in Reacted Xyloglucan -Based Gel

Reacted xyloglucan gel (RXG) was prepared using a method published previously by our research group (Dalvi et al., 2021). Briefly, a 3.0% w/v solution of tamarind seed xyloglucan (TSX) prepared in 10 mM of sodium acetate buffer (pH 5.0) was treated with β galactosidase enzyme obtained from *Aspergillus oryzae* to partially remove galactoside residues. The reaction was kept at 35°C for 18 h. To terminate the reaction, the entire reaction mixture was heated to 90°C for 30 min. RXG was obtained by precipitation using 95 %v/v ethanol followed by drying in an oven at 50°C. The formulation development and optimization were based on several parameters which are discussed in detail in our previously published work (Dalvi et al., 2021). The optimized, freeze-dried Rufi-Ch-NPs were dispersed in a 2.0 %w/v solution of RXG using a magnetic stirrer to form a Rufi-Ch-NPs-loaded reacted xyloglucan-based *in situ* gelling formulation (Rufi-NP-RXG). To the Rufi-NP-RXG formulation, thiomersal was added at a level of 0.01% v/v as a preservative.

### Characterization of Formulations

#### Measurement of Zeta Potential and Particle Size of Rufi-Ch-NPs

Particle size, polydispersity index (PDI), and zeta potential of Rufi-Ch-NPs were measured using a zeta sizer instrument (Nano ZS, Malvern Instruments Ltd., Worcestershire, United Kingdom). The intensity of the scattered light was measured at a backscatter angle of 173°. All measurements were performed at 25°C and an equilibration time of 2 min was set for all samples before the measurements were made.

#### Determination of Entrapment Efficiency and Drug Loading

Entrapment efficiency (EE %) and drug loading were evaluated using an indirect and direct method, respectively. For the indirect method, the nanoparticle suspension was centrifuged at 10,000 × g for 15 min to obtain a pellet. This was washed three times to remove free drug adhering to the surface of NPs. The supernatant was suitably diluted and free drug was analyzed using a previously developed and validated HPLC method. The EE % was calculated using the following formula:

$$EE\%=\frac{W_{total\;Rufi}-W_{free\;Rufi}}{W_{total\;Rufi}}\times 100$$

Where, $W_{total\;Rufi}$ is the amount of Rufi used in the preparation of nanoparticle formulation and $W_{free\;Rufi}$ is the amount of Rufi in the supernatant. In case of the direct method for estimation of EE %, the nanosuspension formulation was washed with three times its volume of water and centrifuged at 10,000 × g. The supernatant was discarded and the pellet obtained was dissolved in a suitable solvent. Further, it was centrifuged at 10,000 × g and the supernatant was diluted and analyzed for Rufi using a previously developed and validated HPLC method (Dalvi et al., 2018).

#### Morphological Analysis of Rufi-Ch-NPs

A scanning electron microscope with a sputter coater (FE-SEM, FEI, Apreo LoVac, TermoFisher Scientific, MA, United States ; EM UC7 Leica Ultra Microtome, Wetzlar, Germany (Sputter Coater)) was used to view the surface and size of the formed nanoparticles. A total of 50 µL of the formulation was spread evenly on an aluminum stub and left to dry under vacuum for 12 h. Sputter coating was achieved with gold under an inert environment. The sputter-coated sample was kept in a vacuum chamber to capture images at an acceleration voltage of 5 kV.

#### Thermal Analysis Using DSC

Differential scanning calorimetric analysis (DSC 60; Shimadzu Corporation, Kyoto, Japan) was performed for Rufi-Ch-NPs and components. Briefly, weighed samples (5 mg) were taken in aluminum pans and crimp sealed. The thermograms were obtained in the temperature range of 10–300°C with a heating rate of 10°C/min in an inert N_2_ environment.

### Rheological Evaluation of the Formulations

Rheological evaluation (Anton Paar MCR 302, Graz, Austria) was carried out to determine the T_sol→gel_ (solution to gel transition temperature) for Rufi-NP-RXG. The LVER (linear viscoelastic region) was identified using an amplitude sweep. Measurements were performed in the oscillatory mode using a temperature sweep within the LVER of the sample. The change in storage modulus (G′) with change in temperature was plotted for both Blank-RXG and Rufi-NP-RXG.

### *In vitro* Drug Release Study From Rufi-NP-Susp and Rufi-NP-RXG Formulations

*An in vitro* drug release study was carried out by a membrane-less sample and a separate method for the aqueous suspension of Rufi-Ch-NPs (Rufi-NP-Susp) and Rufi-NP-RXG formulations. Dissolution was carried out in 250 ml beakers, all of the same dimensions. At the base of the beaker, an aluminum pan was glued at the center. The beakers were pre-equilibrated at 34 ± 1°C for around 30 min. Formulation quantity equivalent to 2.5 mg of Rufi was carefully dropped into the aluminum pan which was glued to the beaker. Within 2 min of addition of the formulations, 125 ml of dissolution medium, [simulated nasal electrolyte solution (SNES)] pre-equilibrated at 34 ± 1°C, was carefully poured into the beakers. The beakers were left in the incubator orbital shaker at 34°C, 100 rpm. Samples of 2 ml were withdrawn at predetermined time points (60, 120, 240, 360, 480, 720, and 1,440 min) from each beaker at different time points. The samples were centrifuged at 11,269 × g for 30 min at 10°C. Samples were analyzed using a validated RP-HPLC analytical method (as given in chapter 2). The results from the *in vitro* study were fitted into different mathematical models viz. zero order, first order, Higuchi, and Korsmeyer-Peppas models. The mechanism of drug release was determined based on the value of ‘*n*’ obtained from the Korsmeyer-Peppas model. Similarity factor (f2) was used for pair-wise comparison of the dissolution profiles of Rufi-NP-Susp and Rufi-NP-RXG formulations.

### Stability of Rufi-Ch-NPs

Stability was checked for both Rufi-Ch-NPs and Rufi-NP-RXG. Freeze-dried Rufi-Ch-NPs were stored separately in airtight vials (*n = 3*) at room temperature conditions (25 ± 2°C and relative humidity of 60 ± 5%), and Rufi-NP-RXG was stored separately in refrigerated conditions (2–8°C). Samples were collected after every 15 days and evaluated for their PS, EE %, zeta potential, and drug content.

### *In-Vivo* Studies in Male Wistar Rats

*In vivo* studies included the assessment of nasal mucociliary transit time and pharmacokinetic (PK) evaluation of Rufi-NP-RXG and an aqueous suspension of Rufi-Ch-NPs (Rufi-NP-Susp). Adult male Wistar rats weighing 240–260 g were used for these studies. Animals were housed in our institute’s animal housing facility which is maintained at controlled temperature, humidity, and light-dark cycle (22 ± 1°C, 55 ± 10% relative humidity, and 12 h light-dark cycle). After procuring the animals, they were allowed to acclimatize to the new environment for at least 10 days before using them for experimentation. Food and water were provided *ad libitum;* except during experimentation, food was not provided until 4 h post-dosing; water was provided during experimentation. All animals were kept for overnight fasting before carrying out PK studies. Prior approval was obtained from the institute’s animal ethics committee (approval number: BITS: Hyd/IAEC/2017/19) for all the procedures carried out during animal experimentation.

#### Nasal Dose Administration and Dosage Precision

The set-up and the technique used for nasal administration of the dose were optimized in our previously published work (Dalvi et al., 2021). A total of 10 µL of the formulation was administered in one nostril of the animals using a pipette and cannula-microtip set-up while maintaining the animal under isoflurane anesthesia. After administering the dose, the animal was kept in a supine position until it recovered from the anesthesia.

A dosing precision study was carried out for Rufi-NP-RXG and Rufi-NP-Susp. Using the abovementioned set-up for nasal dosing, 10 µl of the formulation was pipetted out and analyzed for Rufi after suitable dilutions. This was repeated six times for each formulation, and the relative standard deviation (RSD %) for the amount of Rufi delivered each time was calculated.

#### Mucociliary Transit Time of Formulations

MTT for RXG-Rufi-Ch-NPs and an aqueous suspension of Rufi-Ch-NPs was measured as per the method followed in our previously published work (Dalvi et al., 2021). After administering the formulations as per the abovementioned technique, the oropharyngeal cavity of rats was swabbed with cotton buds at pre-determined time points until 360 min. Rats were not allowed access to food and water until 2 h after administering the dose. Swab samples were analyzed for Rufi using a previously developed and validated HPLC method. The time point at which Rufi was detected in the swab samples was recorded for each animal for both formulation groups. The study was done in triplicate (*n = 3 animals for each formulation).*

#### Pharmacokinetic Studies

Plasma and brain distribution PK studies were performed for Rufi-NP-RXG and Rufi-NP-Susp. The results from these studies were compared with an aqueous suspension of Rufi (Rufi-Susp) (data from previous work) (Dalvi et al., 2021). In the study, for both the treatments, a formulation equivalent to 1 mg/kg of Rufi dose with a dose volume of 40 ml/kg was administered to the rats. A retro orbital puncture technique was used to withdraw blood samples at pre-determined time points: pre-dose, 5, 15, 30, 45, 60, 120, 240, 360, 480, and 600 min. Around 200 µl of blood was collected at each time point in centrifuge tubes containing 4.5 %w/v solution of EDTA sodium salt (EDTA solution was used at 10% v/v of blood collected).

Brain tissue from animals was harvested at pre-determined time intervals post-dosing: 30, 60, 120, 240, and 480 min (*n = 4* animals were used at every time point). Plasma and brain samples were processed using a previously developed and validated RP-HPLC method (Dalvi et al., 2018). PK parameters were computed using Phoenix WinNonlin software version 8.1 by employing NCA analysis of the data obtained from the PK studies.

#### Quantification of Direct Nose to Brain Uptake of Formulation

Two parameters *viz.* % DTE (direct transport efficiency) and % DTP (nose to brain direct transport percentage) were computed in order to quantify and compare the brain uptake of Rufi-NP-RXG and Rufi-NP-Susp formulations.

$$\%DTE=\left(\frac{{\left(AUC_{brain}/AUC_{blood}\right)}_{in}}{{\left(AUC_{brain}/AUC_{blood}\right)}_{i.v}}\right)\times 100$$ (2)

$$\%DTP=\frac{B_{i.n}-B_{x}}{B_{i.n}}\times 100$$ (3)

Where,

$$B_{x}=\frac{B_{i.v}}{P_{i.v}}\times P_{i.n}$$ (4)

Where $B_{i.n}$ is the brain AUC_0→tlast_ following i.n. administration of a given formulation; $B_{i.v}$ is the brain AUC_0→tlast_ following i.v. administration of Rufi; $P_{i.n}$ is the blood AUC_0→tlast_ following i.n. administration of a given formulation; $P_{i.n}$ is the blood AUC_0→tlast_ following i.v. administration of Rufi, and B*x*is the brain AUC_0→tlast_ fraction contributed by the distribution from systemic circulation through the BBB following i.n. administration of the developed formulations. The higher the value for DTE, the better the reach of the formulation to the brain. The higher the %DTP value, the greater the uptake of formulation via nose to brain pathways.

### Statistical Evaluation of Data

To determine statistically significant/critical variables affecting the response variables, analysis of variance (ANOVA) was performed for the data obtained from the experimental runs conducted based on the Minires screening design. In the case of the optimization design using the BBD, the regression model between each of the response variables and their corresponding critical factors were tested and validated based on various diagnostic plots. In addition, the significance of the regression model for each of the response variables $\mathrm{PS}\left(Y_{1}\right)$, zeta potential $\left(Y_{2}\right)$, and EE % $\left(Y_{3}\right)$ were evaluated based on the results obtained from ANOVA, adjusted *R* ^2^, and predicted *R* ^2^ values.

In the case of the *in vivo* PK studies and *in vitro* characterization, the data were expressed as mean ± SD. One-way ANOVA was used to compare PK data obtained from different experimental groups at a 5% level of significance. If the ANOVA results showed a statistically significant difference, a suitable post hoc test was applied to further compare the groups.

## Results

### Preliminary Trials and Screening for Critical Factors Using Minires Design

From the preliminary trials, a total of six independent factors/variables were identified with their upper and lower limits. A screening design was performed to select the statistically significant critical factors affecting the response variables (PS, EE %, and zeta potential).

Out of the six factors, the main effect of four factors *viz.* X_1_-chitosan: STPP mass ratio, X_2_-volume of PEG, X_3_-amount of poloxamer 407, and X_4_-homogenization speed was found to be statistically significant. Statistical analysis of the model revealed that the regression models obtained in the screening design were significant for all three responses. The ‘F_Cal_’ values for regression models of the responses were 8.33 (*p < 0.05)* for PS, 312.2 (*p < 0.001)* for zeta potential, and 934.15 (*p < 0.001)* for EE %. In addition, the ANOVA results revealed that the curvature was significant in all three regression models.

### Optimization of Critical Factors for the Preparation of Rufi-Ch-NPs

The BBD, being one of the two popular quadratic response surface methods, was selected for the optimization of the response variable as a function of the four critical factors. The sum of squares, ‘F_Cal_’ value, and ‘*P*’ value for the factorial term are given in Table 1. The factors with ‘*P*’ values less than 0.05 were considered to have a statistically insignificant effect on the response. Table 1 also shows the ‘F_Cal_’ values for lack of fit, pure error, and model terms for each response. Factors affecting individual response variable and their regression equation are discussed in the subsequent sections.

**TABLE 1:** Statistical output (ANOVA) for optimization model for all responses.

Particle size (Y_1_)				Zeta potential (Y_2_)				Entrapment efficiency (Y_3_)			
Significant terms	SS	df	*p value*	Significant terms	SS	df	*p value*	Significant terms	SS	df	*p value*
Model	1.483E^+007^	4	<0.0001	Model	2697.21	4	<0.0001	Model	212.69	2	<0.0001
X_1_	9.427E^+006^	1	<0.0001	X_1_	1675.60	1	<0.0001	X_1_	24.60	1	0.0365
X_2_	142.83	1	*0.6263	X_2_	20.54	1	*0.1520	X_1_^2^	188.09	1	<0.0001
X_1_ X_2_	9438.12	1	0.0006	X_1_ X_2_	57.002	1	0.0217				
X12	5.390E^+006^	1	<0.0001	X_1_^2^	944.06	1	<0.0001				
Residual	12,885.57	22		Residual	205.23	22		Residual	92.97	21	
Lack of fit	12672.85	20	#0.1533	Lack of fit	182.59	20	#0.6894	Lack of fit	68.59	19	#0.9170
Pure error	212.72	2		Pure error	22.65	2		Pure error	24.37	2	
Total	1.484E+007	26		Total	2902.45	26		Total	333.06	26	

SS- sum of squares; # non-significant lack of fit; * even though the *p* value is more than 0.05, these terms are included in the model to preserve the hierarchy of the model; df- degrees of freedom.

#### Effect of Critical Factors on Particle Size

The least square polynomial equation (in terms of coded factors) describing the effect of critical factors on PS (after ignoring insignificant factors) at 95% confidence level is as follows:

$$PS\left(Y_{1}\right)=232.48-886.35X_{1}+3.45X_{2}+48.58X_{1}X_{2}+899.15X_{1}^{2}$$ (5)

**FIGURE 1:**
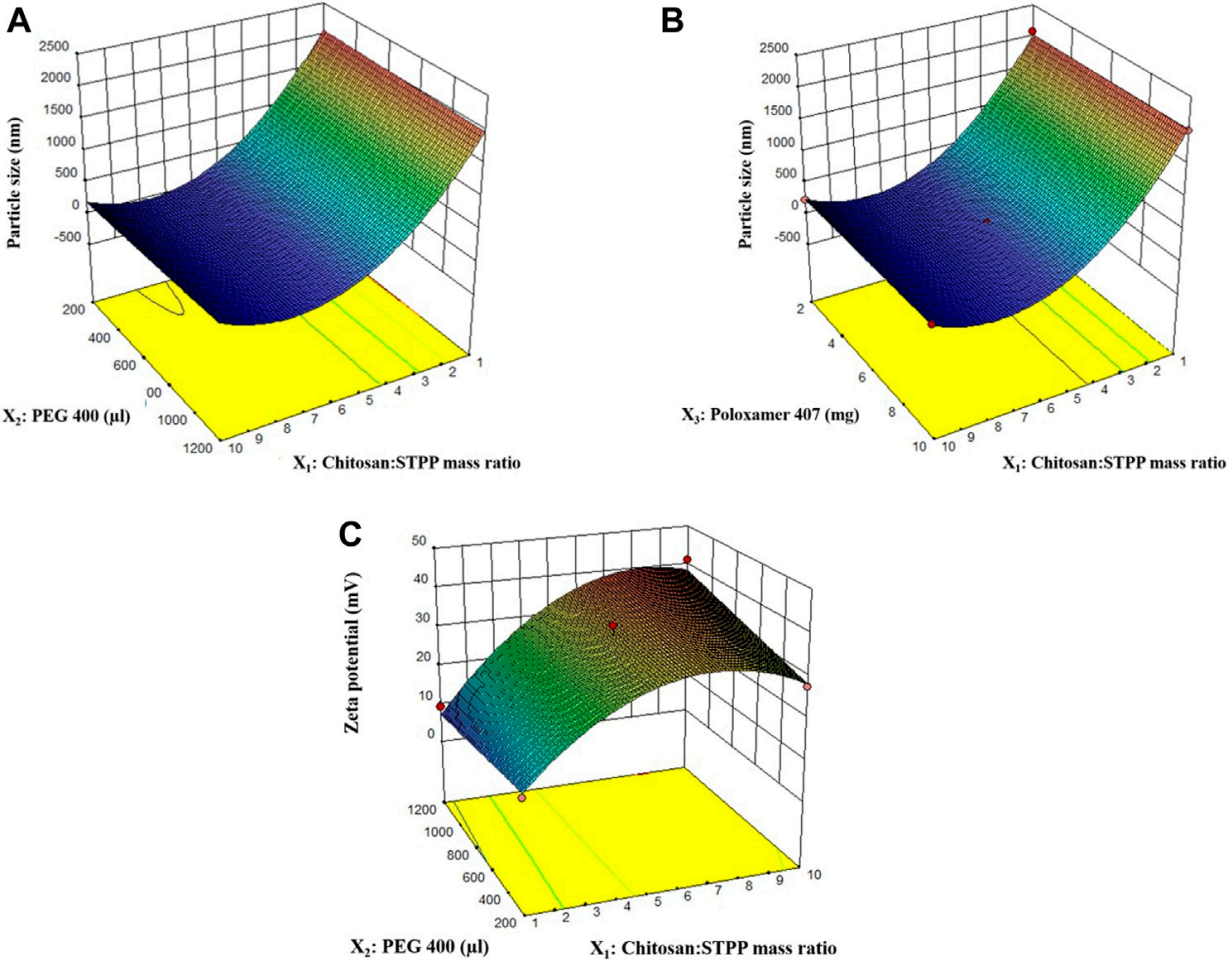
3D response surface plots for effect of factors on particle size **(A, B)**, and zeta potential **(C)**.

A quadratic model was suggested by the software for PS. The model was found to have an ‘F_Cal_’ value of 6328.59 with *p < 0.0001*. The lack-of-fit was insignificant (*p > 0.05*). The predicted *R* ^2^ value was 0.9985 and the adjusted *R* ^2^ value was 0.9990. The difference between the two values was less than 0.2 which is indicative of the closeness of observed and predicted responses by the model equation for PS. The distribution of residuals was random around zero with no specific pattern. All these indicated that the chosen model fitted well to the response variable, PS. Highest PS was observed for the 9^th^ run with a PS of 2077.1 nm and the smallest PS was observed for the 22nd run with a PS of 179.9 nm. The effect of the chitosan:STPP mass ratio and volume of PEG 400 on PS is shown in Figure 1A.

#### Effect of Critical Factors on Zeta Potential

The least square polynomial equation (in terms of coded factors) describing the effect of critical factors on zeta potential (after ignoring insignificant factors) at 95% confidence level is as follows:

$$\mathrm{ZetaPotential}\left(Y_{2}\right)=32.73+11.82X_{1}+1.31X_{2}+3.78X_{1}X_{2}-11.90X_{1}^{2}$$ (6)

The quadratic model for zeta potential was found to have an ‘F_Cal_’ value of 72.28 with *p < 0.0001*. The ‘*P*’ value for ‘lack-of-fit’ for the model was 0.6894, suggesting that lack-of-fit is insignificant (Table 1). Both these results indicate how well the model fits the data obtained for zeta potential. The distribution of residuals for different runs was randomly distributed around zero with no specific pattern. Higher values of predicted *R* ^2^ (0.8857) and adjusted *R* ^2^ (0.9164) with their difference less than 0.2 indicate that values predicted using the model will be in close agreement with the observed values. Overall, the model diagnostics showed that the model fitted the data well. Chitosan:STPP mass ratio, volume of PEG 400, and their interaction had a significant effect on zeta potential. The effect of chitosan:STPP mass ratio on zeta potential is shown in Figure 1C.

#### Effect of Critical Factors on Entrapment Efficiency

The EE % did not vary significantly for the runs in the design. The EE % varied between 64 and 75% across various experimental runs in the optimization. Based on the results obtained from ANOVA, only chitosan:STPP mass ratio had a significant impact on the EE %. The change in EE % from 64 to 75% is primarily due to the increase in chitosan:STPP mass ratio in those runs. The remaining factors did not have any significant effect on the EE % of the Rufi-Ch-NPs.

#### Desirability Value and Validation of the Model

Desirability is a mathematical method to find the optimum values of experimental conditions in order to achieve the desired responses. A set of experimental conditions (given by the optimization model) having a desirability value close to 1 is chosen as the optimum point in the design space. The desirability value obtained by Design Expert software for the simultaneous optimization of PS and EE % was 0.960. The experimental conditions predicted by the software based on the quadratic models for PS and EE % were: Chitosan:STPP mass ratio- 9.3; volume of PEG 400–1,200 μl; amount of poloxamer 407–3.5 mg; homogenization speed-11473 rpm. The optimized formulation showed a PS of 180 ± 1.5 nm, PDI of 0.29 ± 0.08, zeta potential of 38.3 ± 1.5 mV, EE % of 75 ± 2.0%, and drug loading of 11 ± 0.3%. To check the validity of the model given by the software, six verification runs (*n* = 6) were carried out. Formulations were prepared as per the experimental conditions given by the model and the responses *viz*. PS, zeta potential, and EE % were measured. The observed values for the responses were compared with the predicted values given by the model. The two sets of values were compared using a Wilcoxon signed rank test at a 5% level of significance (α = 0.05). No significant difference between the observed and predicted values was found; PS (*p* = 0.069) and zeta potential (*p* = 0.347).

### Characterization Studies

**FIGURE 2:**
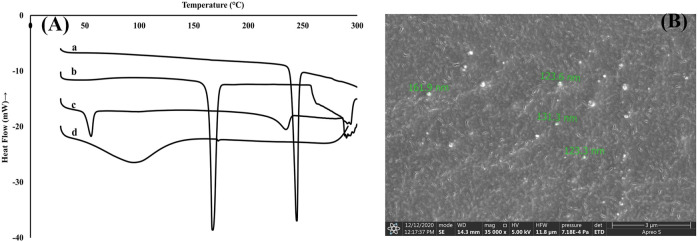
Characterization of Rufi-Ch-NPs **(A)**: DSC thermograms of pure Rufi (a), freeze-dried Rufi-Ch-NPs (b), physical mixture of all components of Rufi-Ch-NPs (c), and pure chitosan (d) **(B)**: Scanning electron microscope image of Rufi-Ch-NPs.

The DSC thermograms are shown in Figure 2A. The thermogram for pure Rufi showed a sharp endothermic peak at 240°C. Pure chitosan showed a broad endothermic peak at 80°C which is attributed to the loss of water molecules from chitosan. There was no change in the characteristic peak of Rufi in the physical mixture, which indicates an absence of incompatibility between Rufi and other excipients used in the preparation of the Rufi-Ch-NPs. The thermogram of freeze-dried Rufi-NP-RXG showed a sharp endothermic peak at 160°C which corresponds to the melting point of mannitol, which was used as the cryoprotectant in the freeze-drying process of Rufi-Ch-NPs. Apparently, the absence of the characteristic peak for Rufi in Rufi-Ch-NPs might be due to its presence in amorphous form in the nanoparticle matrix. The SEM image of optimized Rufi-Ch-NPs is shown in Figure 2B. From the image, it can be seen that the nanoparticles are spherical in nature.

### Rheological Evaluation

**FIGURE 3:**
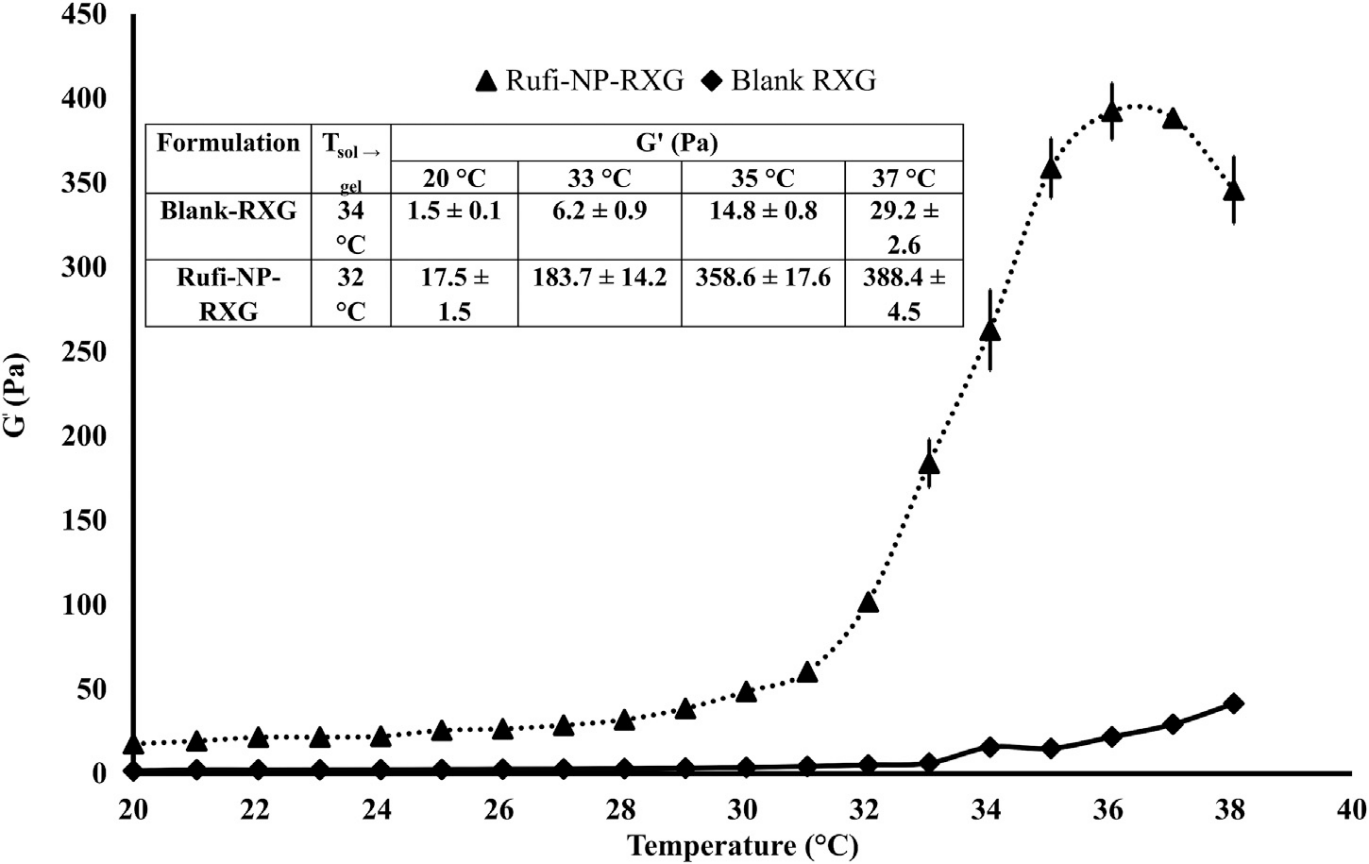
Rheological evaluation for Rufi-NP-RXG and Blank-RXG using temperature sweep. Note: Analysis for each formulation was performed in triplicate. Each data point is represented as mean ± SD of *n* = 3 determinations.

The T_sol→gel_ was determined in the LVER of the formulations. The T_sol→gel_ for Rufi-NP-RXG was compared with the T_sol→gel_ of Blank-RXG (Dalvi et al., 2021). The G′ values for both formulations at different temperatures are given in Figure 3. The plots for G′ values vs temperature for Rufi-NP-RXG and Blank-RXG are shown in Figure 3. The G′ values for Rufi-NP-RXG were consistently higher than Blank-RXG at all temperatures.

### *In Vitro* Drug Release Study From Rufi-NP-Susp and Rufi-NP-RXG Formulations

**FIGURE 4:**
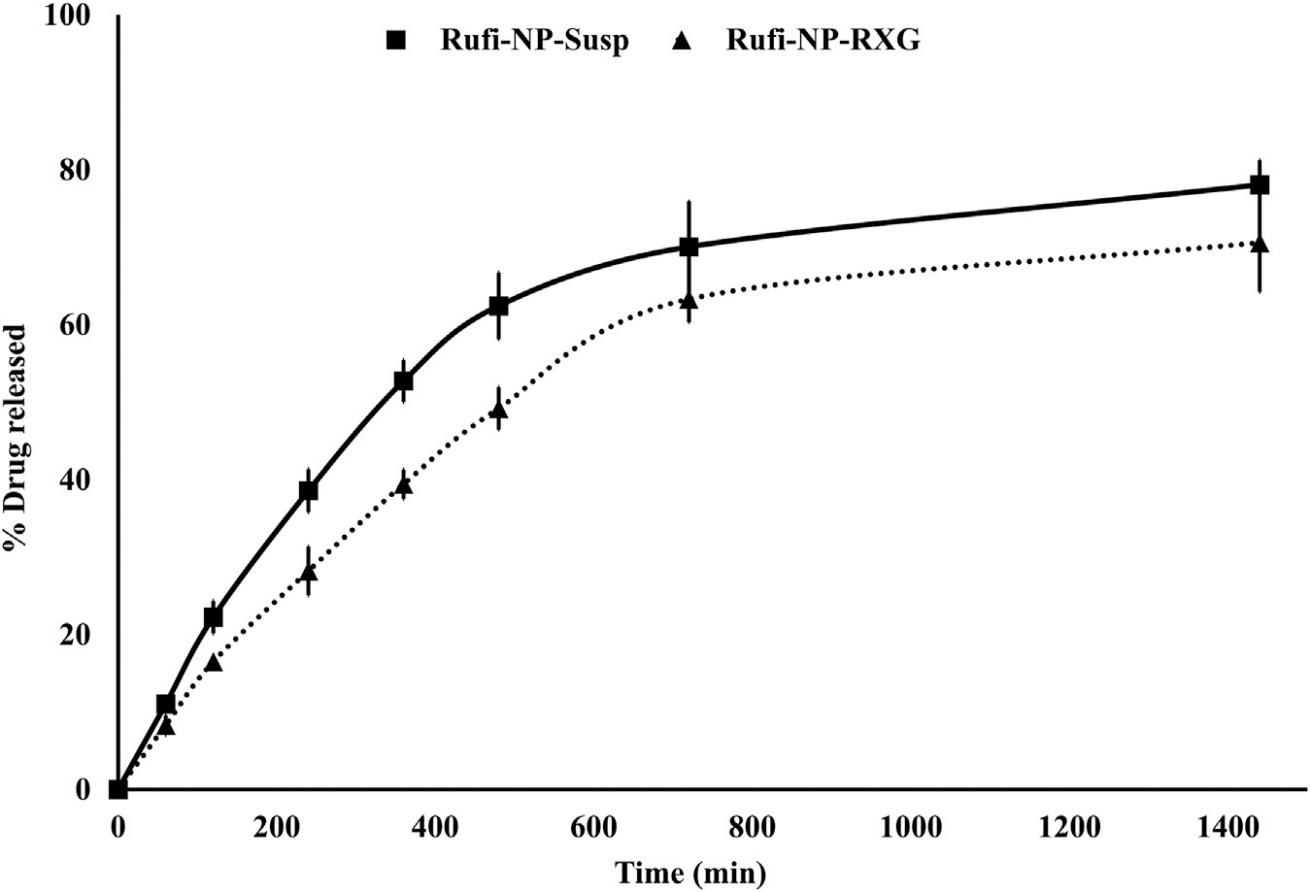
*In vitro* drug release profiles of Rufi-NP-Susp and Rufi-NP-RXG in simulated nasal electrolyte solution (SNES). Note: Each data point is a mean ± SD of three independent observations (*n* = 3).

The *in vitro* release studies were performed to evaluate the kinetics and mechanism of drug release from Rufi-NP-Susp and Rufi-NP-RXG formulations. The release profile is shown in Figure 4. We have modeled the release of Rufi only from the Rufi-NP-Susp formulation, because in the case of the Rufi-NP-RXG formulation, the release of Rufi is affected at two different stages—release of Rufi from the Rufi-Ch-NPs and release of free Rufi from the RXG gel formulation in the dissolution medium. The release profile of Rufi from the Rufi-NP-Susp formulation was fit to different release kinetics models and it was observed that the Higuchi kinetics model (*R* ^2^ = 0.9267) was the best fit model when compared to the zero order kinetics model (*R* ^2^ = 0.7313) and first order kinetics model (*R* ^2^ = 0.8691). The ‘n’ value for the Korsmeyer-Peppas model was 0.64 for Rufi-NP-Susp indicating that the mechanism of drug release followed non-Fickian diffusion.

### Stability of Formulations

Rufi-Ch-NPs and Rufi-NP-RXG formulations did not show a significant difference in their PS, PDI, zeta potential, and %EE for a period of 60 days. The % bias calculated for all the parameters at each interval for both the formulations was found to be not more than 5.0%. This is indicative of the physical and chemical stability of both the formulations. The stability data are given in Table 2.

**TABLE 2:** Stability studies of freeze-dried Rufi-Ch-NPs and Rufi-NP-RXG.

Stability of freeze-dried Rufi-Ch-NPs (stored at 25°C and 60 ± 5%)					
Parameter evaluated	Time (days)				
	0	15	30	45	60
PS (nm)	180 ± 1.5	185 ± 4.5	179 ± 5.3	188 ± 5.1	189 ± 2.8
Zeta potential (mV)	38.3 ± 1.5	39.1 ± 0.94	39.3 ± 2.9	37.3 ± 1.15	39.9 ± 0.9
EE %	75 ± 2	76 ± 2	72 ± 1.5	71 ± 2	73 ± 2
PDI	0.29 ± 0.08	0.28 ± 0.02	0.28 ± 0.03	0.28 ± 0.02	0.31 ± 0.02
Stability of Rufi-NP-RXG (stored at 2–8°C)					
PS (nm)	181 ± 2.3	180 ± 2.9	179 ± 7.4	186 ± 3.1	186 ± 5.7
Zeta potential (mV)	39.1 ± 1.0	40.4 ± 1.0	39.6 ± 0.73	40.8 ± 2.4	37.6 ± 1.98
EE %	72 ± 2.5	72 ± 2.8	73 ± 3.2	74 ± 3	70 ± 2
PDI	0.29 ± 0.02	0.29 ± 0.06	0.29 ± 0.04	0.28 ± 0.05	0.30 ± 0.04

Data are represented as mean ± SD of *n = 3* replicates of each formulation stored at their respective storage conditions.

### *In Vivo* Studies in Male Wistar Rats

#### Nasal Dose Administration and Dosing Precision Studies

Formulations were administered using the microtip-cannula set-up that was designed specifically for delivery of formulations near the olfactory region. The details of the microtip-cannula set-up were reported in our previous research work on *in-situ* gelling systems for N2B delivery (Dalvi et al., 2021). The PK studies for all the formulations were carried out at a drug dose of 1 mg/kg. The maximum dose volume that can be administered to a rat weighing around 250 g is up to 30 µL (120 μl/kg) (Turner et al., 2011). The dose volume for nanoparticle-loaded formulations was optimized at 40 μl/kg per nostril. The possibility of the formulation leaking out of the animal’s nose was ruled out before performing any studies. Prior to *in vivo* studies, *n = 4* animals were dosed with Rufi-NP-Susp mixed with Amaranth dye. To check for any leakage, the nasopalatine duct of the animals was monitored continuously for the appearance of the dye. The dose volume of 40 μl/kg did not cause any immediate leakage from the animal’s nose.

Loading high amounts of solids in any formulation may bring in variability while administering the dose intranasally. Therefore, a dosing precision study was carried out for Rufi-NP-RXG and Rufi-NP-Susp formulations. The RSD (%) values for the amount of Rufi delivered using the nasal delivery set-up for both formulations were less than 10% indicating precise dosing.

#### Mucociliary Transit Time

The mucociliary clearance was measured by a method used in our previously published work (Dalvi et al., 2021). The mucociliary transit times of Rufi-Susp, Rufi-NP-Susp, and Rufi-NP-RXG were 11.6 ± 2.8 min, 33.5 ± 5.7 min, and 53.3 ± 11.5 min, respectively. Statistical comparison of MCC times of Rufi-NP-RXG and Rufi-NP-Susp using a *t*-test showed significant difference (*p* = 0.027) in their MCC time.

#### Pharmacokinetic Studies

**FIGURE 5:**
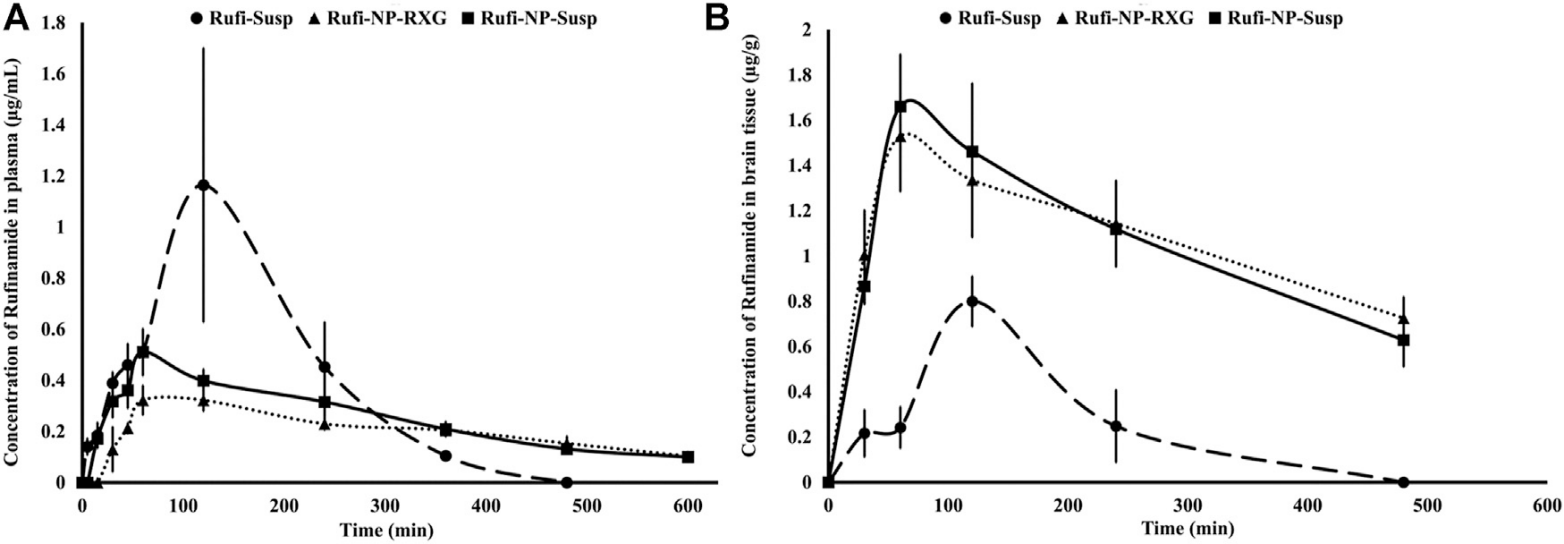
Plasma and brain pharmacokinetic performance of formulations. **(A)**: Mean concentration time profiles of Rufinamide obtained following intranasal administration of Rufi-NP-Susp, Rufi-NP-RXG, and Rufi-Susp in plasma; **(B)**: Mean concentration time profiles of Rufinamide obtained following intranasal administration of Rufi-NP-Susp, Rufi-NP-RXG, and Rufi-Susp in the brain. Note: Each data point is a representation of mean ± SD of *n* = 4 animals for plasma PK, and for brain PK *n* = 4 animals were used at every time point.

Pharmacokinetic studies were performed according to the procedures given in *Pharmacokinetic Studies*. The plasma time course profiles of Rufi-NP-Susp and Rufi-NP-RXG are shown in Figure 5A. We compared the pharmacokinetic performance of Rufi-NP-Susp and Rufi-NP-RXG with that of Rufi-Susp (Table 3) (PK data for Rufi-Susp was taken from our previously published work) (Dalvi et al., 2021).

**TABLE 3:** Pharmacokinetic parameters of Rufi-NP-RXG, Rufi-NP-Susp, and Rufi-Susp in brain and plasma following in. administration at a drug dose of 1 mg/kg.

Matrix	Pharmacokinetic parameter	Units	Treatments		
			Rufi-NP-Susp	Rufi-NP-RXG	Rufi-Susp
Plasma	AUC0_→tlast_	min×(µg/ml)	145.7 ± 21.7	123.1 ± 10.3	200.75 ± 74.4
	C_max_	µg/mL	0.49 ± 0.06	0.35 ± 0.04	1.16 ± 0.53
	T_max_	min	60	90	120
	MRT	min	232.9 ± 3.7	218.4 ± 4.9	147.4 ± 9.4
Brain	AUC0_→tlast_	min×(µg/g)	509.3	512.17	104.28
	C_max_	µg/g	1.68 ± 0.2	1.60 ± 0.2	0.79 ± 0.11
	T_max_	min	60	60	60
	% DTE		988.5	1177.3	146.8
	% DTP		86.06	91.5	31.9

All the parameter values are expressed as mean ± SD of (*n = 4*) animals per treatment group. T_max_ is expressed as a range or a single value wherever applicable; in the case of plasma data, mean AUC_0-tlast_ obtained from four animals (*n = 4*) was used while in the case of brain data, AUC_0-tlast_ obtained from composite sampling with four animals sacrificed at each time point was used.

PK parameters like AUC_0→tlast_, C_max_, T_max_, and MRT were calculated by NCA (non-compartmental analysis) using Phoenix WinNonlin Version 8.1. A one-way ANOVA comparison for plasma AUC_0→tlast_ values for all three formulations showed that there was no statistically significant difference between any of the three formulations. The MRT values of both the nanocrystal formulations were significantly higher than the MRT for Rufi-Susp.

**FIGURE 6:**
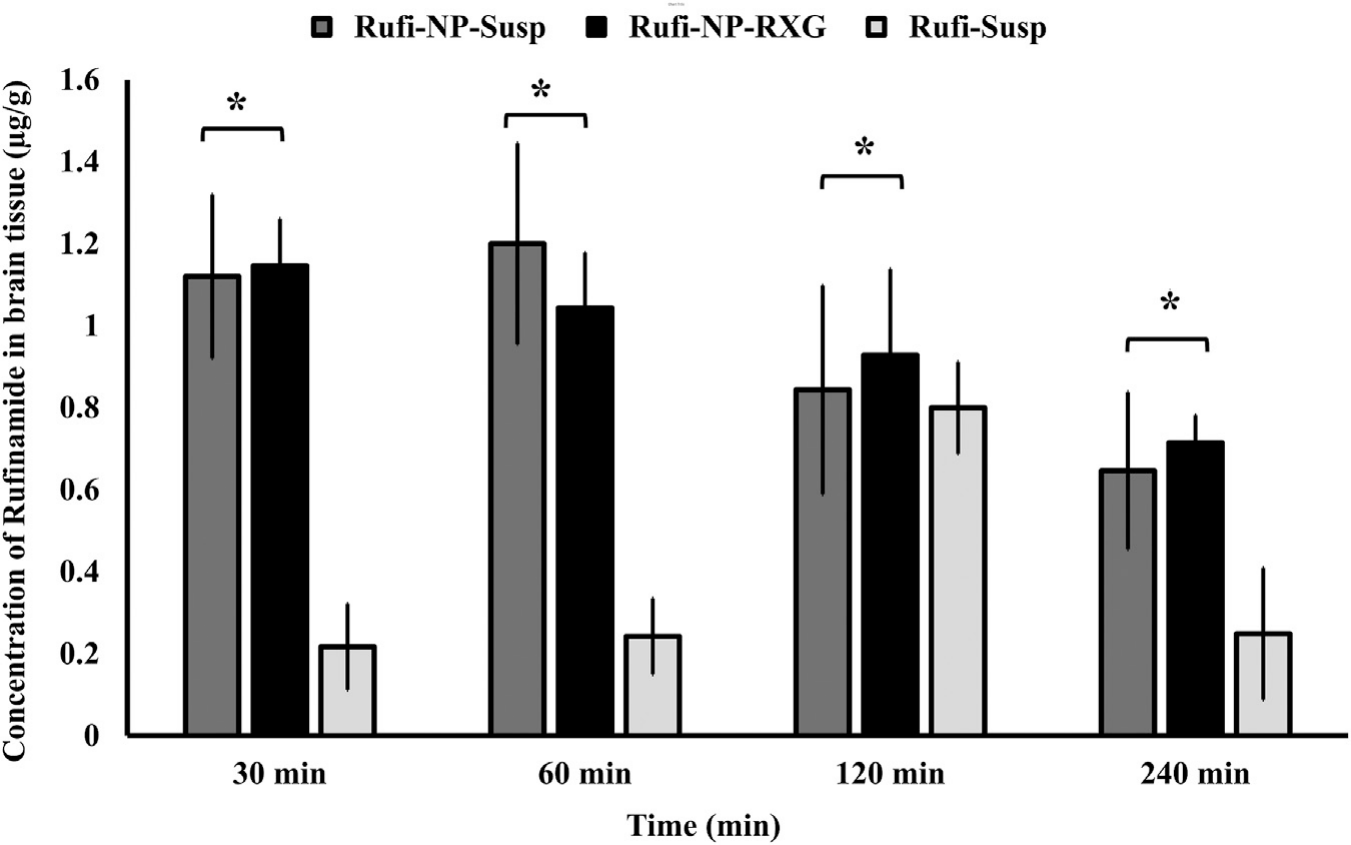
Comparison of concentration of Rufinamide in brain following intranasal administration of Rufi-NP-Susp, Rufi-NP-RXG, and Rufi-Susp. Note: Each bar represents mean ± SD of brain concentrations of n = 4 rats; Statistically no significant difference unless mentioned otherwise; all statistical tests were performed for dose-normalized concentrations using *t*-test at α = 0.05.

The brain PK parameters for all three treatments are shown in Table 3. The brain concentrations vs time profile of Rufi-NP-Susp, Rufi-NP-RXG, and Rufi-Susp are shown in Figure 5B. The brain AUC_0→tlast_ values for both the nanoparticulate formulations were almost five times the brain AUC_0→tlast_ values for Rufi-Susp. Further, the brain concentrations were compared for all three treatments at four different time points- 30, 60, 120, and 240 min using *t*-test at alpha of 0.05 (Figure 6). At each time point for every treatment group, *n =* 4 animals were sacrificed. The concentration of Rufi at each time point was an average of the pooled concentration from four animals, the underlying assumption being that inter-individual differences accounted for residual variability rather than the inherent differences in the PK process of different treatments. The average brain concentration vs time profile could be constructed only from pooled concentration data, and hence, values of brain AUC_0→tlast_ were given as single values without standard deviation. Consequently, ANOVA and other statistical comparison tests could not be applied to brain data unlike plasma data. Instead, the brain concentrations were compared at four different time points for all treatments using *t*-tests.

At all the time points, 30, 60, 120, and 240 min, there was no significant difference between the brain concentrations of Rufi-NP-RXG and Rufi-NP-Susp formulations. However, at all the time points, the brain concentrations of both the nanoparticulate formulations were significantly higher than Rufi-Susp.

To evaluate the performance of formulations further, % DTE and % DTP values were calculated as per Eqs 2, 3, and 4 and are given in Table 3. The DTP values give the percentage of drug taken up directly by the brain via nose to brain pathway. %DTP for Rufi-NP-Susp, Rufi-NP-RXG, and Rufi-Susp were 86.06, 91.5, and 31.9, respectively.

## Discussion

### Preliminary Trials, Screening and Optimization Designs

Prior to the identification of critical factors using a screening design, a few preliminary trials were performed to choose the molecular weight of chitosan and the stabilizer, and also to set the limits for various factors used in the screening design. A few trials were performed with low, medium, and high molecular weight chitosan with the same degree of deacetylation (75–85%). It was observed that at same processing conditions and same concentrations, high molecular weight chitosan consistently yielded nanoparticles with greater PS than medium and low molecular weight chitosan. This may be because of higher viscosity of high molecular weight chitosan than the other two grades, which impeded the diminution of the particles beyond a certain size (12). It was observed that the EE % values with low molecular chitosan were consistently less than 50%. A similar observation was reported by Mohammadreza Abbaspour et al. (13). Out of several stabilizers tested, poloxamer 407 was chosen because it did not increase the solubility of Rufi in the dispersion medium (used in preparation of nanoparticles). PEG 400 was used as a co-solvent to maintain Rufi in the solubilized form during the addition of STPP solution into the Rufi solution during the preparation of nanoparticles.

For the screening of factors identified from the preliminary trials, a Minires screening design with resolution IV was selected. In a resolution IV design, the main effects are not confounded with other main effects or even two factor interactions. The screening design consisted of 17 runs (including 3 center point runs) with each factor at two different levels. Center point runs were performed to determine if the curvature (or the quadratic terms) was significant in the regression model. The information provided by the center point runs (significance of curvature) can help in selecting the appropriate optimization design.

The chitosan:STPP mass ratio greatly affected PS and zeta potential, while the %EE was only slightly affected by it. With increases in chitosan:STPP mass ratio from 1 to 7 units, PS of the nanoparticles decreased significantly. However, as the chitosan:STPP mass ratio is increased beyond 7–10 units, the PS of nanoparticles did not change significantly. This observation was found to be in line with the observations reported by F. Rázga et al. in their review article (Rázga et al., 2016). As the chitosan:STPP ratio decreases below 6, the balance between NH_3_^+^ ions and O^−^ ions shifts toward STPP. For the same amount of STPP, a greater number of NH_3_^+^ groups can bind to the O^−^ ions rapidly. Such a strong ionic interaction hinders further breakdown of particles at the same amount of energy input. No significant change was observed in PS with the change in volume of PEG 400 used in the formulation. Similarly, change in the amount of poloxamer 407 did not have a significant effect on the PS of the nanoparticles (Figure 1B). The effect of homogenization speed on the PS of nanoparticles was also insignificant.

In the case of zeta potential, as the chitosan:STPP mass ratio increased, the zeta potential also increased. This increase in positive zeta potential can be attributed to NH_3_^+^ groups on chitosan which were left unbound from the O^−^ ions of STPP. The interaction term X_1_X_2_ showed a statistically significant effect on the zeta potential. Hence, to maintain hierarchy of the model, the factor X_2_ (volume of PEG 400), although not significant, had to be included. Overall, the zeta potential was profoundly affected by chitosan:STPP mass ratio. The optimized formulations showed a high zeta potential which resulted in strong repulsion, no particle aggregation, and therefore good stability for the formulation.

### Characterization of Rufi-Ch-NPs and Rufi-NP-RXG Formulations

Thermal analysis of freeze-dried Rufi-Ch-NPs showed that there were no interactions that could indicate incompatibility between the components of the formulations. Rheological studies showed that the G′ values for Rufi-NP-RXG were consistently higher than Blank-RXG at all temperatures. This can be attributed to hydrophobic interactions, and some ionic interactions between chitosan and xyloglucan molecules (Martínez-Ibarra et al., 2019). Although the strength of Rufi-NP-RXG (indicated by G′ values) was higher than Blank-RXG even at temperatures below the intranasal temperature, it did not affect the dosing precision of the formulation as indicated in section 3.6.1. Greater strength of Rufi-NP-RXG than Blank-RXG in the gelled state was found to be beneficial in order to retain the formulation in the nasal cavity for a longer time. From the *in vitro* study, the ‘n’ value for the Korsmeyer-Peppas model was 0.64 for Rufi-NP-Susp indicating that the mechanism of drug release followed non-Fickian diffusion, more specifically, a special case of non-Fickian transport–‘anomalous transport’. In anomalous transport, the velocity of solvent diffusion and the polymeric relaxation have similar magnitudes. Around 50% of the drug was released at 360 and 480 min in Rufi-NP-Susp and Rufi-NP-RXG, respectively. At the time point of 1,440 min, Rufi-NP-Susp and Rufi-NP-RXG had released 78 and 70% of Rufi, respectively. A slightly delayed release in the case of the Rufi-NP-RXG formulation may be attributed to a strong cohesive interaction between chitosan chains and RXG chains. The similarity factor (f2) value obtained for the comparison of the drug release profiles of Rufi-NP-RXG and Rufi-NP-Susp (f2 = 52) indicated that their drug release profiles were similar. Stability analysis of both formulations showed that they were stable for a period of 60 days.

### *In Vivo* Studies in Male Wistar Rats

I.n. dosing in rats requires optimization of two aspects *viz.* site of delivery of formulation in the nasal cavity and dose volume. Site specific deposition of formulation into the nose affects the pathway *via* which the drug gets taken up. One of the main strategies to enhance direct nose to brain drug delivery is to deposit the drug at the olfactory region of the nose. Hence a special delivery set-up with a soft and flexible cannula of 1.3 cm attached to a microtip was used to administer formulations. Further, once deposited into the nasal cavity, the formulation should withstand clearance from the nose by the mucociliary clearance (MCC) process. The MCC is a defense mechanism of the nose to push away foreign particles entering the nose. In order to have higher residence time in the nasal cavity, a formulation must resist the MCC process. Rufi-NP-RXG formulations showed a significantly higher MTT compared to other formulations. This can be attributed to the mucoadhesive properties of chitosan arising from an interaction between negatively charged sialic acid residues in mucin and NH_3_^+^ groups of chitosan (Ways et al., 2018).

From the brain pharmacokinetic studies, it was evident that the concentrations of Rufi in the brain were significantly higher in the case of both nanoparticulate formulations compared to the brain concentrations achieved with the Rufi-Susp formulation. Also, the %DTE and % DTP values for both nanoparticulate formulations were significantly higher than Rufi-Susp. This could be attributed to longer residence time of the nanoparticulate formulations inside the nasal cavity. The brain concentrations of Rufi-NP-Susp and Rufi-NP-RXG were not significantly different at any time point, the % DTE and % DTP values were slightly greater for Rufi-NP-RXG formulation. This could be attributed to a slightly higher brain AUC_0→tlast_ value and lower plasma AUC_0→tlast_ value for Rufi-NP-RXG when compared to Rufi-NP-Susp. With the nanoparticles suspended in RXG gel, we expected to observe a significant increase in the brain uptake of nanoparticles. However, the observed PK data (%DTP values and brain concentrations at different time points) of Rufi-NP-Susp and Rufi-NP-RXG formulations did not show a significant advantage of the RXG *in situ* gel in enhancing the brain uptake of Rufinamide. Chitosan shows good mucoadhesive properties, and has been shown to enhance paracellular transport of small molecules, biomolecules, and nanoparticles by altering tight junction proteins. Consequently, a significant amount of Rufinamide brain uptake was observed only with Rufi-NP-Susp alone.

## Conclusion

In this work, the nose to brain uptake of nano formulations of Rufinamide was assessed. Rufinamide-loaded chitosan–STPP nanoparticles were prepared using an ionic gelation technique and optimized based on the principles of DoE. Nanoparticles were characterized for their particle size, zeta potential, and entrapment efficiency. The optimized nanoparticles were loaded in a thermoresponsive nasal *in situ* gel based on modified tamarind seed polysaccharide as the thermoresponsive polymer. *In vivo* plasma and brain pharmacokinetic evaluation was performed for aqueous suspension of nanoparticles, and nanoparticles dispersed in the *in situ* gel. The results were compared with the previously published data for the aqueous suspension of Rufinamide. Data comparison revealed that both the nanoparticle formulations showed better direct nose to brain uptake than aqueous suspension of Rufinamide. This was evident from higher %DTP values obtained for both nanoparticle formulations. Chitosan nanoparticles of Rufinamide significantly enhanced its brain uptake via the direct nose to brain pathway. Given its low oral bioavailability and high dose and dosing frequency, an intranasal nanoparticulate formulation of Rufinamide would make its therapeutic regimen less complicated; especially so when Rufinamide is prescribed to manage Lennox Gastaut Syndrome in patients from ages 1 to 18.

## Data Availability

The raw data supporting the conclusions of this article will be made available by the authors, without undue reservation.
